# Utilization of community-based health planning and services compounds in the Kintampo North Municipality: a cross-sectional descriptive correlational study

**DOI:** 10.1186/s12913-017-2622-4

**Published:** 2017-09-26

**Authors:** Kenneth Wiru, Akwasi Kumi-Kyereme, Emmanuel N. Mahama, Seeba Amenga-Etego, Seth Owusu-Agyei

**Affiliations:** 1 Kintampo Health Research Centre, Ghana Health Service, P.O Box 200, Kintampo, B/A Region Ghana; 2 Department of Population and Health, University of Cape Coast, Cape Coast, Ghana

**Keywords:** Community-based health planning and services, Geographic access, Rural communities, Health service utilization, Community health officers, Ghana, Kintampo North Municipality

## Abstract

**Background:**

The Community-based Health Planning and Services (CHPS) initiative was introduced to improve coverage and utilization of basic health services for people in remote rural communities whose use of orthodox health services was hitherto limited by distance. To achieve this aim, the scheme has so far been scaled up to several communities nationwide as part of government’s agenda to improve the general wellbeing of the populace. The objectives of this study were to examine the extent of patronage of CHPS compounds in the Kintampo North Municipality, factors associated with their use and challenges faced by community members regarding the use of these facilities.

**Methods:**

We adopted a descriptive cross-sectional correlational design for this study. We collected data from 171 household heads or their representatives, selected through a multistage sampling technique. The respondents were drawn from five randomly selected communities among those with CHPS compounds and their proportions weighted based on the populations of these communities.

**Results:**

Our analysis revealed that a high proportion (73.7%) of the respondents patronized CHPS compounds for health care. We also found sex and income to predict the use of the facilities though income was less significant after adjusting for sex in a multivariate analysis. Females were about six times more likely than males to patronize CHPS compounds (adjusted OR = 5.98, 95% CI 2.55, 14.0, *P* = < 0.01). Household heads earning between GH¢ 200.00 and GH¢ 300.00 were about nine times more likely to use the facilities than those who earned below GH¢ 100.00 (adjusted OR = 8.88, 95% CI 1.94, 40.6, *P* = 0.05). Our findings also showed that shortage of medicines (41.5%), lack of money to pay for services (28.7%) and absenteeism of Community Health Officers (CHOs) (12.3%) were major barriers to the use of the facilities.

**Conclusions:**

Based on the foregoing findings, there is an apparent need to ensure timely replenishment of medicines at the facilities and step up supervision of CHOs in order to sustain patronage of the compounds.

**Electronic supplementary material:**

The online version of this article (10.1186/s12913-017-2622-4) contains supplementary material, which is available to authorized users.

## Background

Globally, grave public health concerns like morbidity and health inequity result from disparities in the availability, access and utilization of health services especially in low and middle income countries. It is established that rural dwellers are more restricted in their use of formal sector health services than their urban counterparts in most of these countries [1] due to numerous socio-economic and health system factors. Shortage of skilled personnel, medicines, inadequate personal incomes and long distance to health facilities amongst others have been reported to hamper health service utilization particularly in developing countries [2–4]. For example, Bour [4] reported an inverse relationship between distance and health service utilization in the Ashanti region of Ghana whereas Tsegay and colleagues [5] acknowledged that proximity of villages to health facilities is a strong predictor of the utilization of antenatal health services in Ethiopia. It is also documented that inadequate use of health services diminishes expectation of life and increases infant deaths in rural populations [6].

To ease the adversities associated with underutilization of health services and improve population health, most developing countries have resorted to community-based health schemes. These strategies are expected to engender and accelerate social and economic development through improved utilization of health services in view of the strong nexus between health and development. A number of studies have demonstrated the impact of community health interventions on coverage and utilization of health services worldwide. A classic case in point is the finding from the Ghana Essential Health Interventions Program (GEHIP) that was executed in the Upper East region of Ghana. The GEHIP study resulted in a 100% coverage of essential health services in the intervention districts compared to 50% coverage in the comparison districts [7]. Furthermore, a community-based health services program in the Philippines has been reported to significantly increase the proportion of pregnant women receiving tetanus toxoid injections in an intervention community from 58% in the pre-intervention period to 81% at post-intervention, whilst the proportion of expectant mothers who made at least 3 prenatal visits in the intervention districts increased from 41% to 89% [8]. Moreover, Macinko and Guanais [9] documented in Brazil a 10% increase in service coverage, which correlated with a 5% reduction in infant mortality in a community-based Family Health Program. These findings generally attest to the capacity of community oriented health initiatives to improve the utilization of health services in the beneficiary communities and general population wellbeing.

In Ghana, efforts to improve coverage and utilization of basic health services through local community mobilization date back to the colonial era [10]. However, virtually all these initiatives were short-lived due to resource and organizational constraints. Even so, there were renewed attempts to provide community-level vital health services to the populace after the declaration of the Primary Health Care (PHC) initiative in 1978. Based on evidence from the Danfa Comprehensive Rural Health and Family Planning Project and the Brong Ahafo Regional Integrated Development Project in the mid 1970s, the Government of Ghana deployed trained village health workers in rural communities to render essential health services [11].

Furthermore, government piloted the United Nations Children’s Fund (UNICEF)-sponsored Bamako Initiative which was designed to optimize health services access and use in 1987. However, this scheme was deserted for reasons akin to those that caused the demise of previous health service strategies. Precisely, rapid volunteer attrition, poorly trained personnel and financial bottlenecks effectively ruined this initiative [12]. Subsequently, Community Health Nurses were trained and stationed in health centres to treat minor ailments and provide periodic community outreach services with a concentration on maternal and child health. But the rural outreach programmes of these nurses were fraught with transportation and resource difficulties thereby rendering them underutilized and ineffective.

Failure of the foregoing strategies to improve access and use of health services spurred the quest for a robust health system, which culminated in the development and implementation of the community-based health planning and services (CHPS) strategy in 2000 [13]. Modelled on the concept of PHC, CHPS is intended to enhance coverage and utilization of essential health services for populations in rural settings [13]. Under this initiative, districts are divided into sub-districts and further split into zones with service populations of between 3000 and 4500 in order to ensure efficient planning and delivery of health services. In each zone, a resident Community Health Officer provides both mobile doorstep and facility-based health services as well as collaborates with a Community Health Committee and health volunteers to plan and execute community-based health programmes [11, 14]. Consequently, the country’s health service has been structured into a three-tier system with the community health compound being the first point of an individual’s contact with the health system [11, 15].

Since the inception of this scheme, it has been scaled up to 3175 functional CHPS zones and 1410 functional CHPS compounds nationwide whilst efforts are being made to create more zones and make them conterminous with local electoral areas in the country to boost health service delivery [13].

This paper explored the extent of patronage of CHPS compounds in the Kintampo North Municipality and discussed factors and barriers associated with their use.

### Conceptual framework

The conceptual framework for this study is based on Andersen and Newman’s behavioural model of health service utilization. The model posits that health service use is a function of predisposing, enabling and need factors. It essentially intends to link people’s personal characteristics to the use of health services [16]. Predisposing factors are crucial determinants of the likelihood of need for health care. They are demographic characteristics such as age, gender, marital status, level of education among others, which vary amongst persons. Hence, individuals with certain kinds of demographics may have different health needs or consume more health services than others with differing demographic characteristics although these features do not directly cause the need to utilize health services [16].

Enabling factors are the means available to aid access to and use of health services. They are community-level resources such as health facilities, health personnel and quality of health services available to people in their residential and occupational locations [16]. If a community has many health facilities and clients do not have to queue for long hours in order to obtain services, people may use them more frequently than in areas with fewer facilities and throngs of people competing for services. Personal enabling resources that determine access to and use of health services are income and wealth that allow individuals to pay for health services as determined by their health insurance status and government’s policy on cost-sharing regarding the use of health services [16].

Need factors are the most direct causes of health service utilization [16]. They are either perceived or evaluated as the model stipulates. Perceived need entails how people view their general health and functional state as well as their experience of symptoms of disease or pain and whether they deem these conditions to be of adequate seriousness to require the use of health services [16]. Conversely, evaluated need, is the professional assessment of a person’s health status and need for health care. In keeping with this model, perceived need better explains an individual’s utilization behaviour since the decision to use a service is partly driven by perception of need [16]. For example, the occurrence of an illness or injury which creates discomfort or pain for an individual can lead him or her to seek professional help. Hence, the higher the severity or frequency of an illness the more likely a person is to use health services. Perceived need is therefore a significant factor to investigate in studies of health service utilization [16]. Even though the model encompasses several individual determinants, just a number of them have been included in the conceptual framework as not all such variables can be investigated within the ambit of the current analysis.

**Fig. 1:**
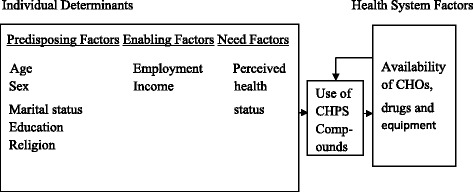
Conceptual Framework for Studying the Utilization of CHPS Compounds. Source: Adapted from Andersen and Newman (2005)

Figure 1 represents the conceptual framework adapted for studying the utilization of CHPS compounds. Individual determinants and health system factors are independent variables that influence the use of CHPS compounds (a visit to a CHPS Compound by a household head to seek care for himself/herself or for his/or her child), which is the dependent variable. It is believed that all the independent variables interact to determine initial use of health services from CHPS compounds. However, subsequent visits to a CHPS compound may be a function of health system enabling factors. This is due to the fact that clients would most likely form impressions about the nature of services provided in the compounds after their initial visit. Thus, their decision to make subsequent and repeated visits to the compounds might be determined by their perception of service quality, availability of community health officers and medicines (Fig. 1).

## Methods

### Study area

The Kintampo North Municipality is one of the twenty-seven administrative districts of the Brong Ahafo Region of Ghana [17]. Geographically, it is located between latitudes 8°10’ and 8°65′ North of the equator and Longitudes 1°35′ and 2°00̕’ West of the Greenwich meridian. The Kintampo Township marks the central point between the northern and southern parts of the country. The municipality is bounded to the north by the Central Gonja District and to the south by the Kintampo South District. Westwards, it is bounded by the Wenchi, Tain and Bole Districts of the Brong Ahafo and Northern Regions respectively whilst it is bounded to the East by the Pru and East Gonja Districts. It covers an area of about 5108 km^2^ representing 12.9% of the total land area of the region with a population of 95,480 [17].

The area is inhabited by two major indigenous ethnic groups - Bono and Mo who reside in their respective traditional communities with most of the Bono communities situated in the Eastern and South-Eastern parts of the municipality whilst the Mo predominantly occupy its Western corridor. Aside these two indigenous ethnic groups, the area has many immigrant populations of northern descent as well as a few Ewes and Dangbes who have settled along the Black Volta River where they fish for their livelihood. Its economy is largely agrarian with the major produce being yam, maize, cassava and plantain. Much of it is rural with roads that are hardly usable in the rainy season.

**Fig. 2:**
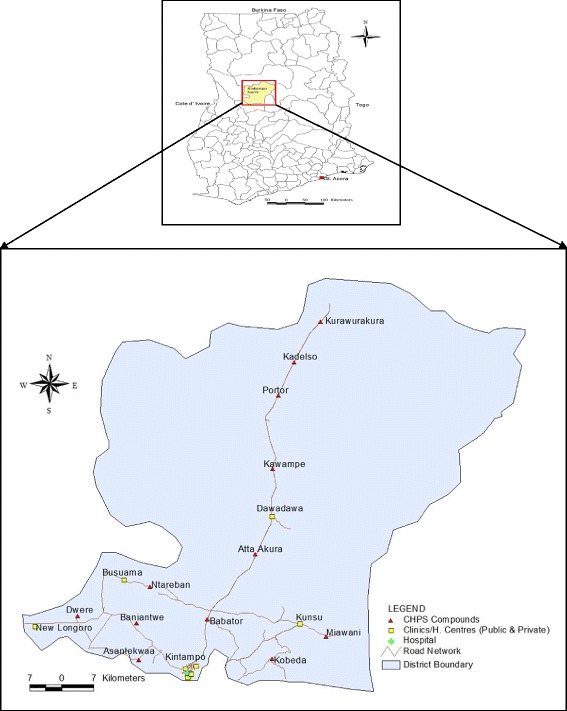
Map of the Kintampo North Municipality showing Health Facilities. Source: Geographic Information Systems’ Unit, Kintampo Health Research Centre (2016)

The Municipality has a general hospital in Kintampo from which people access health care. There are also four private health facilities, two public rural clinics, two health centres, one outreach office and twelve functional CHPS compounds across the municipality to provide for the health needs of the inhabitants (Fig. 2).

### Study design

We adopted a descriptive cross-sectional correlational design for this study. This design was adopted because of its advantage of allowing for the collection of data from a sample population at one point in time and description of relationships between study variables of interest [18].

### Sampling procedure

There were twelve functional CHPS communities in the Kintampo North Municipality at the time of this study. These communities had a total of 3427 active residential compounds. We employed a multistage sampling technique in selecting study participants in order to ensure representativeness. The twelve CHPS communities formed the first stage sampling units from which we randomly sampled five using Stata SE 12. We randomly selected 5 % of the active residential compounds (171 compounds) from these five CHPS communities with their proportions weighted based on the number of compounds in each community. One household head or his/her representative was conveniently sampled in each of the sampled residential compounds bringing the total number of respondents to 171. This is in keeping with the suggestion that a sample of 100 respondents is the least number required for descriptive studies whilst a sample of at least 50 is needed to establish the existence of relationships in correlational studies [19].

### Data collection method

Data was collected through the administration of an interview schedule (Additional file 1) to respondents by trained field supervisors. This technique was employed because of its advantage of allowing interviewers to clarify difficult questions and probe deeper for clear-cut responses [20]. The interview schedule covered questions on background characteristics of respondents, use of CHPS compounds and barriers to their use.

### Data management and analysis

We carefully checked all forms for inconsistencies before data entry and analysis. The data was entered in FoxPro version 9 and exported to Stata SE 12 for statistical analysis. We generated descriptive statistics from the data to describe background characteristics of respondents, extent of utilization of CHPS compounds and barriers to their use. Variables that were categorical in nature were summarized as proportions whilst continuous variables were expressed as means. We used univariate and multivariate logistic regression models to determine relationships between the explanatory variables of interest and use of CHPS compounds (the outcome variable). Our explanatory variables were sex, marital status, age, level of education, employment status, income level and religion.

## Results

### Background characteristics of study participants

Of all the 171 respondents interviewed, the mean age was 36.8 years with a standard deviation of 12.9. The minimum age of the respondents was 18 years whilst the maximum age was 80 years. A few (3.5%) of the respondents were under 20 years of age. Majority (59.1%) of them ranged between 20 and 39 years of age. Female participants were 67.3% whereas the remaining 32.7% comprised of males.

Over half of the respondents (58.5%) were Muslims whilst Christians constituted 36.2%. Practitioners of traditional religious beliefs constituted 2.3% of the respondents whereas 3% of them belonged to other religious groupings. Majority of them (65%) had no formal education. Respondents with primary education were 17.5% whilst those who attained middle or junior high school were 11.7%. The least proportion (5.8%) of the respondents attained secondary or higher education (Table 1).

**Table 1:** Background Characteristics of Respondents

Characteristic	Frequency (*n* = 171)	Percentage (%)	
Age			
< 20	6	3.5	
20–39	101	59.1	
40–59	52	30.4	
60+	12	7.0	
Mean age = 36.8	Standard deviation = 12.9	Lowest age = 18	Highest age: 80
Sex			
Male	56	32.7	
Female	115	67.3	
Religion			
Christianity	62	36.2	
Islam	100	58.5	
Traditional religion	4	2.3	
Other	5	3.0	
Level of education			
None	111	65.0	
Primary	30	17.5	
Middle/JHS	20	11.7	
Secondary/Higher	10	5.8	
Marital status			
Single	36	21.0	
Married	118	69.0	
Divorced	6	3.5	
Widowed	11	6.5	
Occupation			
Unemployed	12	7.0	
Farming	102	59.7	
Trading	45	26.3	
Government work	3	1.7	
Other activities	9	5.3	
Monthly income			
Less than GH¢100.00	67	39.2	
GH¢100.00 - GH¢200.00	52	30.4	
GH¢200.00 - GH¢300.00	27	15.8	
GH¢300.00 and above	25	14.6	

GH¢ 3.215 = $ 1.00

Source: Field data, (2014)

More than two-thirds (69%) of them were married. Single respondents were 21% whilst 6.5% of them were widowed. The remaining 3.5% were divorced (Table 1). Most (93%) of the respondents were economically active and engaged in a number of productive ventures. Over half of the respondents (59.7%) were engaged in farming. A number of them (26.3%) were also engaged in diverse trading activities. Relatively fewer respondents (1.7%) were employed in the public sector whereas 5.3% of them were engaged in other economic ventures. Regarding incomes, majority (39.2%) of the sample earned less than one hundred Ghana cedis monthly. Those who earned between One Hundred and Two Hundred Ghana Cedis were 30.4% whilst 15.8% of them earned between Two Hundred and Three Hundred Ghana Cedis per month. Relatively 14.6% of the respondents earned a monthly income above Three Hundred Ghana cedis (Table 1).

### Extent of patronage of CHPS compounds

Our findings indicate that 73.7% of household heads patronized CHPS compounds. We however observed differences in their patronage by background characteristics. As Table 2 depicts, a high proportion (83.3%) of household heads aged less than 20 years patronized the compounds whilst a relatively low proportion (58.3%) of them aged sixty years and above reportedly used the facilities. It is further observed that majority (81.7%) of female household heads patronized CHPS compounds compared with 57.1% of their male counterparts. Besides, 77.4% of Christian household heads used the compounds compared with 75% of believers of traditional faith and 72% of Muslim household heads.

**Table 2:** Extent of Patronage of CHPS Compounds by Background Characteristics of Respondents

Characteristic	Percentage		
	Yes (73.7%)	No (26.3%)	Total (100%)
Age			
< 20	83.3	16.7	3.5
20–39	74.3	25.7	59.1
40–59	75.0	25.0	30.4
60+	58.3	41.7	7.0
Sex			
Male	57.1	42.9	32.7
Female	81.7	18.3	67.3
Religion			
Christianity	77.4	22.6	36.3
Islam	72.0	28.0	58.5
Traditional religion	75.0	25.0	2.3
Others	60.0	40.0	2.9
Education			
None	72.1	27.9	64.9
Primary	90.0	10.0	17.5
Middle/JHS	60.0	40.0	11.7
Secondary/Higher	70.0	30.0	5.9
Marital status			
Single	72.2	27.8	21.0
Married	75.4	24.6	69.0
Divorced	66.7	33.3	3.5
Widowed	63.6	36.4	6.5
Employment			
Unemployed	66.7	33.3	7.0
Farming	71.6	28.4	59.7
Trading	80.0	20.0	26.3
Government work	100.0	0.0	1.7
Others activities	66.7	33.3	5.3
Income			
< GH¢100.00	64.2	35.8	39.2
GH¢100.00 - GH¢200.00	7.5	2.5	3.0
GH¢200.00 - GH¢300.00	88.9	11.1	15.8
GH¢300 and above	80.0	20.0	14.6

GH¢ 3.215 = $ 1.00

Source: Field data, (2014)

Majority (90%) of respondents with primary education patronized CHPS compounds whilst 72.1% of those without formal education used them. Similarly, 70% of respondents with secondary or higher education patronized CHPS compounds compared with 60% of those with middle or junior high school education (Table 2).

Additionally, the bivariate analysis showed that a greater proportion (75.4%) of married household heads patronized CHPS compounds. A high proportion (72.2%) of single household heads also patronized the facilities compared with divorced and widowed household heads who reported patronage levels of 66.7% and 63.6% respectively (Table 2).

Close to three-quarters (74.2%) of household heads in employment utilized CHPS compounds compared with 66.7% of their unemployed counterparts. Similarly, approximately 89% of the respondents who earned between two hundred and three hundred Ghana cedis monthly patronized the compounds. This contrasts with 75% of household heads earning between one hundred and two hundred Ghana cedis and 64.2% of those earning less than one hundred Ghana cedis monthly (Table 2).

### Factors associated with the utilization of CHPS compounds

Among our explanatory variables, sex and income emerged as significant determinants of utilizing CHPS compounds. None of the other variables was significant in the univariate analysis (Table 3). The odds ratios showed that females were a little over three times more likely to use CHPS compounds than males (OR = 3.35, 95% CI 1.63, 6.90, *P* = 0.01). Household heads who earned between GH¢ 200.00 and GH¢ 300.00 monthly were also about four times more likely to use a CHPS compound than those who earned less than GH¢ 100.00 (OR = 4.46, 95% CI 1.18, 16.7, *P* = 0.02).

**Table 3:** Univariate and Multivariate Logistic Regression Analyses of Factors Influencing the Use of CHPS Compounds

	Univariate logistic regression				Multivariate logistic regression		
Characteristics	n (%)	OR^ρ^	95% CI	P^α^	OR^ϕ^	95% CI	P^β^
Age							
< 20	5 (83.3)	1					
20–39	75 (74.3)	0.84	(0.15, 4.38)	0.83	–	–	–
40–59	39 (75)	0.90	(0.15, 5.18)	0.91	–	–	–
60+	7 (58.3)	0.28	(0.03, 2.18)	0.22	–	–	–
Gender							
Male	32 (57)	1			1		
Female	94 (81.7)	3.35	(1.63, 6.90)	0.01	5.98	(2.55, 14.0)	< 0.01
Religion							
Christianity	48 (77.4)	1					
Islamic	72 (72)	0.75	(0.35, 1.58)	0.45	–	–	–
Traditional	3 (75)	0.87	(0.08, 9.43)	0.91	–	–	–
Other religious beliefs	3 (60)	0.43	(0.06, 2.87)	0.38	–	–	–
Educational level							
None	80 (72)	1					
Primary	27 (90)	3.48	(0.97, 12.41)	0.05	–	–	–
Middle/JHS	12 (60)	0.58	(0.21, 1.57)	0.28	–	–	–
Secondary/Higher	7 (70)	0.90	(0.21, 3.76)	0.89	–	–	–
Marital status							
Single	26 (72.2)	1					
Married	89 (75.4)	1.18	(0.50, 2.75)	0.70	–	–	–
Divorced	4 (66.7)	0.76	(0.11, 5.00)	0.78	–	–	–
Widowed	7 (63.4)	0.67	(0.15, 2.84)	0.58	–	–	–
Employment							
Unemployed	8 (66.7)	1					
Farming	73 (71.6)	1.25	(0.34, 4.56)	0.72	–	–	–
Trading	36 (80)	2	(0.48, 8.17)	0.33	–	–	–
Government work	3 (100)	1	–	–	–	–	–
Other activities	6 (66.7)	1	(0.15, 6.28)	1.00	–	–	–
Monthly income level							
< GH¢100.00	43 (64)	1			1		
GH¢100.00–200.00	39 (75)	1.67	(0.74, 3.78)	0.21	2.16	(0.88, 5.27)	0.08
GH¢200.00–300.00	24 (88.9)	4.46	(1.18, 16.7)	0.02	8.88	(1.94, 40.6)	0.05
GH¢300 and above	20 (80)	2.23	(0.73, 6.77)	0.15	5.41	(1.62, 18.0)	0.06

GH¢ 3.215 = $ 1.00

Source: Field data, (2014)

OR^p^: Unadjusted odds ratio CI: Confidence interval P^α^: *P*-value

OR^ϕ^: Adjusted odds ratio P^β^: Adjusted P-value

Moreover, the association between sex and CHPS compound use became more significant after adjusting for income in the multivariate analysis. Females were about six times more likely to use a CHPS compound than males (adjusted OR = 5.98, 95% CI 2.55, 14.0, *P* = <0.01). However, income appeared less significant after adjusting for sex in the multivariate analysis (Table 3). Household heads earning between GH¢ 200.00 and GH¢ 300.00 were about nine times more likely to use the facilities than those who earned below GH¢ 100.00, though with a borderline *P*-value (adjusted OR = 8.88, 95% CI 1.94, 40.6, *P* = 0.05) (Table 3).

### Barriers to the use of CHPS compounds

Shortage of medicines in CHPS compounds was reported by 41.5% of the respondents (37.5% of males and 43.5% of females respectively) as a major barrier (see Table 4). Besides shortage of medicines, a relatively high proportion (28.7%) of the respondents mentioned inadequate money to pay for services. However, men appeared more financially challenged than women as 37.5% of them reported their inability to use the facilities due to lack of income whereas 24.4% of their female counterparts cited same reason for not patronizing the compounds. Furthermore, 12.3% of household heads (8.9% of males and 13.9% of females respectively) stated that absenteeism of Community Health Officers made it difficult for them to utilize health services (Table 4).

**Table 4:** Barriers to the Use of CHPS Compounds

Barrier	Male	Female	Total
	n (56) %(32.7)	n (115) % (67.3)	n (171) % (100)
Lack of money	37.5	24.4	28.7
Shortage of medicines	37.5	43.5	41.5
Bad attitude of CHO	0	6.1	4
CHO not available	8.9	13.9	12.3
Long waiting times	1.8	4.3	3.5
Don’t know	14.3	7.8	10

Source: Field data, (2014)

## Discussion

Overall, our findings have shown a high patronage of CHPS compounds by community members in the study area. Although the current study did not measure the relationship between distance and patronage of the compounds, the evidence garnered suggests that the high patronage of the facilities stems from their proximity to community members. This is due to the fact that most people in CHPS beneficiary communities do not have to travel longer distances to access health care as distance has been documented in previous studies to negatively affect health service use [3, 4]. The high patronage of CHPS compounds also suggests that most persons perhaps lacked a convenient source of care prior to the introduction of the CHPS strategy. This implies that access to locally available basic health services may reduce the use of high order and distant facilities, which inhabitants of rural and low-income areas may find difficult to access.

The observed age variations in patronage of the compounds, albeit less striking suggests a downward trend in their use with progression in age. This seems to contradict the notion that frailty and need for health care become grave as people age and thus trigger increased consumption of health services among the aged. However, the trend observed in this study could either result from a lack of enabling resources for the aged to use health services rendered in the compounds (Fig. 1) or from the inclusion of many young women of reproductive age in the study, who most likely sought care for their children and also used antenatal and postnatal services. Provided that the former scenario holds true, our result contrasts findings of previous research that suggest a positive association between age and health service utilization [21] whereas it confirms the finding that proximity of villages to health facilities strongly encourages the use of antenatal services if the latter is true [5]. We also observed religious variations in patronage of the compounds though an association was not established between religious affiliation and use of the facilities. This notwithstanding, Christians were found to patronize the compounds most; thereby confirming an earlier finding that Ghanaian Christians utilize more modern health services than their Muslim counterparts [22].

Furthermore, our findings underscore the positive predisposing effect of sex on health service utilization [16]. The finding that more women patronized CHPS compounds and were more likely to use them than men could be due to the fact that women have higher morbidity and use more gynaecological and obstetric services particularly in their child bearing ages [23]. The availability of maternal and child health services in CHPS compounds therefore explains the high female patronage. Conversely, the relatively low level of patronage among men possibly arises from the lack of special health services targeting men’s health in these compounds. Consequently, our findings are consistent with findings of other studies that showed a higher patronage of health services for women than men [24, 25].

Additionally, our bivariate analysis indicates that patronage of CHPS compounds increases with educational attainment. This is probably because educated persons have better knowledge of health risks, benefits and places where health services can be obtained thereby making them more inclined to use services. Lack of knowledge about treatments obtainable from health facilities due to deficient education could therefore prevent uneducated persons from using them and worsen their disease burden. This finding compares with findings of previous research in Ghana and Senegal that showed that education positively impinges on the use of health services [22, 26]. Also, our results indicate that patronage of CHPS compounds is to some extent influenced by marital status (Fig. 1). The higher patronage of the facilities among married persons is perhaps due to the economic support that individuals in marriage unions benefit from each other. However, our finding contrasts the result of Prior and Hayes [27] that suggests that married persons use health facilities less than unmarried persons.

Analysis of the extent of patronage of the facilities by employment status showed a higher rate of patronage among employed participants than the unemployed. This finding implies that employed persons are better able to pay for health services from CHPS compounds due to the incomes earned from their jobs. Consequently, our findings validate the enabling role of employment on health service utilization reported in other studies [28, 29]. However, the role of employment in utilizing CHPS compounds becomes more evident from a close examination of the rates of patronage by type of employment of household heads. The finding that all (100%) household heads in public sector employment and four-fifths of traders patronized CHPS compounds apparently results from the fact that their jobs provide them with income on a regular basis and so they are able to afford health services from the compounds any time they have need for care. Conversely, the relatively low level of patronage of the facilities by farmers might stem from their irregular incomes as they might be unable to afford the cost of services each time the need for care arises considering the seasonal nature of their trade.

We have effectively shown that income has an enabling effect on the patronage of CHPS compounds in the study area. Our findings imply that use of CHPS compounds increases with rising incomes. Even so, we observed that a number of household heads in the highest income category (GH¢300.00 and above) probably used health services from other facilities as they could afford the cost of transport to distant high order facilities. This is because their patronage of the compounds was about 9% lower than the level of patronage for household heads in the Two Hundred to Three Hundred Ghana Cedis income bracket. Our finding is therefore consistent with the findings of Jatrana and Crampton [30] and Blackwell and colleagues [31] which illustrate statistically significant relationships between gender, income and health service utilization.

Ultimately, the study revealed that shortage of medicines, inadequate income and absenteeism of Community Health Officers in CHPS compounds are major impediments to the use of the facilities. These findings generally parallel those of prior studies in utilization literature and the real challenges that led to the demise of similar public health initiatives in the past. Classic examples of such programs are village health posts under the Primary Health Care initiative in Ghana and Community Health Clinics in Bangladesh [11, 32]. Our finding that absenteeism of CHOs impeded the use of the compounds is due to the fact that some of them do not reside in the CHPS compounds as expected.

Nevertheless, the findings emphasize the significance of health system and personal enabling resources to the use of health services (Fig. 1). Moreover, our finding that more men were financially restricted from using the compounds contrasts findings from a Chinese study that suggest that men utilize more health services and spend more on health care than women as they are financially better off [33]. Indeed, men may have the resources to pay for health care but feel more reluctant than women to seek care even if there is need for care due to cultural reasons.

### Limitations

Considering time and financial inadequacies, it was not feasible to use a more representative sample of the population than was used in this study as larger numbers of respondents would have been required to be interviewed. Thus, this may reduce the reliability of our findings since the results might not have enough power to be generalised to the studied population. A larger study will be desirable to confirm some of the results in this study.

Additionally, our study was mainly based on self-reported information on utilization of CHPS compounds which may be difficult to validate. In view of the disjointed nature of health records obtained from the facilities, we could not extract data on patronage to complement that which was elicited from the field survey. The inclusion of quality data from these records in the analysis could have therefore provided a clear-cut picture on the extent to which the compounds were patronized.

## Conclusions

Our study shows that CHPS compounds are highly patronized in the study area. Hence, they are achieving the main purpose for which the CHPS initiative was designed and implemented. Predisposing and enabling factors (sex and income) are significant determinants of the use of these compounds, though sex is a better predictor of utilization than income. Shortage of drugs, absenteeism of Community Health Officers and financial difficulties of community members are the main challenges which must be adequately addressed in order to sustain and/or improve patronage of the facilities.

## Additional file

Additional file 1: Interview schedule for data collection on the utilization of Community-based Health Planning and Services (CHPS) Compounds (A list of questions asked to study participants). (DOCX 37 kb)

## Abbreviations

CHO: Community Health Officer
CHPS: Community-based Health Planning and Services
GEHIP: Ghana Essential Health Interventions Program
PHC: Primary Health Care
UNICEF: United Nations Children’s Fund
