# Supplementary material for: Utilization of community-based health planning and services compounds in the Kintampo North Municipality: a cross-sectional descriptive correlational study
Source: BMC Health Serv Res. 2017 Sep 26;17:679. doi: 10.1186/s12913-017-2622-4 (PMC5615810; doi:10.1186/s12913-017-2622-4)
Supplement: Additional file 1: — Interview schedule for data collection on the utilization of Community-based Health Planning and Services (CHPS) Compounds (A list of questions asked to study participants). (DOCX 37 kb) [file 12913_2017_2622_MOESM1_ESM.docx]

SUPPLEMENTARY FILE 1

INTERVIEW SCHEDULE FOR DATA COLLECTION ON THE UTILIZATION OF CHPS COMPOUNDS IN THE KINTAMPO NORTH MUNICIPALITY

INTRODUCTION: This interview is to enable KHRC collect information on the utilization of CHPS Compounds in the Kintampo North Municipality. Your participation in this study will be much appreciated; but you are free to opt out if you do not want to participate. All the information volunteered would be kept confidential and anonymized in order to safeguard your identity and responses provided for the study.

|  |  |  |  |  |  |  |
| --- | --- | --- | --- | --- | --- | --- |
|  | | | | | | |
|  |  |  |  |  |  |  |
|  |  |  |  |  |  |  |

Village code: VCODE

Village Name: VNAME

Compound ID: COMPID

FS code: FSCODE

**SECTION A: BACKGROUND CHARACTERISTICS OF STUDY PARTICIPANTS**

1. Age of respondent

|  |  |
| --- | --- |

RAGE

2. Gender

| 1 | Male | 2 | Female |
| --- | --- | --- | --- |

RSEX

| 1 | Christianity | 2 | Islamic | 3 | Traditional | 4 | Other (specify) |
| --- | --- | --- | --- | --- | --- | --- | --- |

3. What is your religion?

RELIG

4. What is the highest level of education you have attained?

| 1 | None | 2 | Primary | 3 | Middle/JSS | 4 | Secondary/higher | 5 | Other (specify) |
| --- | --- | --- | --- | --- | --- | --- | --- | --- | --- |

REDU

5. What is your ethnicity?

| 1 | Mo | 2 | Bono | 3 | Other (specify) |
| --- | --- | --- | --- | --- | --- |

ETHNIC

| 1 | Single | 2 | Married | 3 | Divorced | 4 | Separated | 5 | Widowed |
| --- | --- | --- | --- | --- | --- | --- | --- | --- | --- |

6. What is your marital status?

MARISTAT

7. What work do you do?

| 1 | Unemployed | 2 | Farming | 3 | Trading | 4 | Government work | 5 | Other (specify) |
| --- | --- | --- | --- | --- | --- | --- | --- | --- | --- |

EMPLOY

8. What is your monthly income level?

| 1 | less than GH¢100 | 2 | GH¢ 100-200 | 3 | GH¢ 200-300 | 4 | GH¢ 300 or more |
| --- | --- | --- | --- | --- | --- | --- | --- |

MINCOME

**SECTION B: UTILISATION OF CHPS COMPOUNDS**

9. Have you ever visited the CHPS compound in this community for health services?

| 1 | Yes | 2 | No |
| --- | --- | --- | --- |

EVERVISIT

**If question 9 is yes, ask 10. Otherwise, code NA and proceed to 11**

10. What is the reason for visiting the CHPS compound for health care?

| 1 | Proximity to residence | 2 | Services are good | 3 | Services cost less | 4 | Other (Specify) |
| --- | --- | --- | --- | --- | --- | --- | --- |
| 8 | Don’t Know | 9 | NA |  |  |  |  |

REASUSE

11. What is the reason for not visiting the CHPS compound for care?

| 1 | No health problem | 2 | No money  To pay for services | 3 | Other (specify) | 8 | Don’t Know | 9 | NA |
| --- | --- | --- | --- | --- | --- | --- | --- | --- | --- |

REASNOT

**Ask question 12 if respondent has ever visited CHPS compound for care. Otherwise, code NA**.

12. How many times did you visit the CHPS compound for care in the past one year?

| 1 | Once | 2 | Twice | 3 | Three or more times | 8 | Don’t Know | 9 | NA |
| --- | --- | --- | --- | --- | --- | --- | --- | --- | --- |

NUMVISITS

13. Has any member of your household ever visited the CHPS compound for health care?

| 1 | Yes | 2 | No | 8 | Don’t Know |
| --- | --- | --- | --- | --- | --- |

HMEMVISIT

**Code NA for 14 and 15 and proceed with question 16 if respondent or household member never used CHPS compound.**

14. Do you and members of your household currently use the CHPS compound any time you/they have need for health care?

| 1 | Yes | 2 | No | 8 | Don’t Know | 9 | NA |
| --- | --- | --- | --- | --- | --- | --- | --- |

CURRENTUSE

**If answer to 14 is No, ask 15. Otherwise, code NA and proceed to 16.**

15. What is the reason why you or your household members do not currently use the CHPS compound for health services?

| 1 | Lack of  money | 2 | Poor services  in CHPS compound | 3 | Other (specify) | 8 | Don’t  Know | 9 | NA |
| --- | --- | --- | --- | --- | --- | --- | --- | --- | --- |

WHYNOTCUR

| 1 | CHPS compound | 2 | Spiritualist | 3 | Traditional healer | 4 | Sought no care/ self treatment | 5 | Other (Specify) |
| --- | --- | --- | --- | --- | --- | --- | --- | --- | --- |

16. With regard to the most recent time you were ill, where was your first point of contact for care?

FIRSTCONT

**If no care was sought, code NA for Q17 and proceed to Q18.**

17. Why did you seek care from the facility mentioned?

| 1 | Proximity to residence | 2 | Services are good | 3 | Services cost less | 4 | Other (Specify) |
| --- | --- | --- | --- | --- | --- | --- | --- |
| 8 | Don’t Know | 9 | NA |  |  |  |  |

WHYCARE

18. Why did you not seek care?

| 1 | Lack of  money | 2 | Poor services  in CHPS compound | 3 | Other (specify) | 8 | Don’t  Know | 9 | NA |
| --- | --- | --- | --- | --- | --- | --- | --- | --- | --- |

WHYNOCA

**SECTION C: BARRIERS TO THE USE OF CHPS COMPOUNDS**

19. Are the health needs of you and/or your household members met each time you/they visit the CHPS compound for care?

| 1 | Yes | 2 | No | 8 | Don’t Know |
| --- | --- | --- | --- | --- | --- |

NEEDSMET

20. If answer to 19 is no, probe why? Otherwise, write NA and proceed.

| 1 | CHO not available when needed | 2 | No essential drugs | | 3 | No requisite  equipment | 4 | Other (specify) |
| --- | --- | --- | --- | --- | --- | --- | --- | --- |
| 8 | Don’t Know | 9 | NA |  |  |  |  |  |

WHYNOT

21. In your opinion, what challenges do people face regarding the use of the CHPS compound in this community?

**(Code as many options as provided).** CHAS

| 1 | Lack of money to pay for services | 2 | Shortage of essential medicines | 3 | Unfriendly attitude of CHO |  |  |
| --- | --- | --- | --- | --- | --- | --- | --- |
| 4 | CHO not available when needed | 5 | Long waiting times | 7 | Other (specify) | 8 | Don’t Know |

22. During the past one year, did you or any of your household members have difficulty getting treatment from the CHPS compound?

| 1 | Yes | 2 | No | 8 | Don’t Know |
| --- | --- | --- | --- | --- | --- |

DIFTREAT

| 1 | Lack of money to pay for services | | | 2 | No required drugs | 3 | Long waiting time to see CHO | | |
| --- | --- | --- | --- | --- | --- | --- | --- | --- | --- |
| 5 | CHO not available | 6 | Other (specify) | | | | | 9 | NA |

23. If answer to 22 is yes, ask, what was this difficulty?

DIFICULTY

**Code NA for question 24 and proceed if answer to question 23 is category 2 (No required drugs)**.

24. During the past one year, were you or any of your household members prescribed a medicine but were unable to get it from the CHPS compound?

| 1 | Yes | 2 | No | 8 | Don’t Know | 9 | NA |
| --- | --- | --- | --- | --- | --- | --- | --- |

NOMED

25. In general how would you rate the CHO’s attitude towards his/her clients in this community?

| 1 | Very good | 2 | Good | 3 | Moderate | 4 | Bad | 5 | Very bad |
| --- | --- | --- | --- | --- | --- | --- | --- | --- | --- |

CHOATTIT

26. How does his/her attitude affect the use of the CHPS compound?

| 1 | Encourages use of services | 2 | Discourages use of services | 8 | Don’t Know |
| --- | --- | --- | --- | --- | --- |

ATAFFECT

27. Are there any socio-cultural issues that hinder the use of health services from the CHPS compound in this community?

| 1 | Yes | 2 | No | 8 | Don’t Know |
| --- | --- | --- | --- | --- | --- |

SOCCISUES

**If answer to question 27 is yes, ask 28. Otherwise write NA and proceed.**

28. What are these issues?

| 1 | Religious beliefs | 2 | Traditional beliefs | 8 | Don’t Know | 9 | NA |
| --- | --- | --- | --- | --- | --- | --- | --- |

ISSUES

**SECTION D: SUGGESTIONS TO IMPROVE HEALTH SERVICES FROM CHPS COMPOUNDS**

29. In your opinion, what can be done to improve the delivery and use of health services from CHPS compounds?

**Probe for respondent’s views on measures to be taken by the CHO, District Health Administration or government; and community members to improve the use of the CHPS compound.**

29. (a) What specifically would you want the CHO to do in order to improve the use of the CHPS

Compound by community members?

| 1 | Be regularly available |
| --- | --- |
| 2 | Be respectful to clients |
| 3 | Visit clients at home |
| 4 | Avoid favouritism |
| 5 | Be tolerant to clients |
| 8 | Don’t know |

CHOMEAS

29. (b) What do you think government can do to improve the use of the CHPS compound in this community?

| 1 | Upgrade the CHPS compounds to a health centres |
| --- | --- |
| 2 | Provide the CHPS compound with more drugs |
| 3 | Provide the CHPS compounds with a midwives |
| 4 | Motivate CHO to stay in community |
| 8 | Don’t know |

GOVMEAS

29. (c) What can community members do to improve their use of the CHPS compound?

| 1 | Report problems to health authorities |
| --- | --- |
| 2 | Provide communal support to CHO |
| 3 | Patronize CHPS compound when sick |
| 8 | Don’t know |

COMMEAS

THE INTERVIEW IS ENDED. THANK YOU VERY MUCH FOR YOUR TIME AND COOPERATION
